# Standard Elixirs, with Numerous Formulæ

**Published:** 1874-07

**Authors:** 


*AMERICAN JOURNAL OF PHARMACY—February*, 1874:

STANDARD ELIXIRS.—* * * Prof. Maisch called the attention of the meeting to the circular issued by the American Pharmaceutical Association, containing the report of the Committee on Elixirs, John F. Hancock, chairman; and he urged the adoption of the formulas in order to secure uniformity. An extract of the report, giving the formulas, will be found below:

COMPOUND POWDER OF COCHINEAL.

| | |
|---|---|
| Take of Cochineal in powder, . . . | 120 grains. |
| Alum, in powder, . . . . | 120 grains. |
| Carbonate of Potassium, . . | 120 grains. |
| Bitartrate of Potassium, . . | 240 grains. |

Mix. Keep in well-stopped vial.

COMPOUND TINCTURE OF COCHINEAL.

| | |
|---|---|
| Take of Compound Powder of Cochineal, . | 120 grains. |
| Diluted Alcohol, . . . | 2 fl. ounces. |

Slightly warm the diluted alcohol, and mix with the powder; macerate in a stoppered vial for twelve hours, and filter for use. This is permanent, and imparts a beautiful red color to elixirs and solutions which have no acid properties.

SPIRIT OF ORANGE.

| | |
|---|---|
| Take of Oil of Sweet Orange, . . | 1 fluid ounce. |
| Stronger Alcohol, . . . | 16 fluid ounces. |

Mix. This is made in proportions to conform with the spirits of the U. S. P., and is a pleasant and convenient form of orange flavor.

SIMPLE ELIXIR.

| | | |
|---|---|---|
| Take of | Spirit of Orange, . . . | $\frac{1}{2}$ fluid ounce. |
| | Stronger Alcohol, . . . | 4 fluid ounces. |
| | Cinamon Water, . . . | 6 fluid ounces. |
| | Syrup, . . . . . | 6 fluid ounces. |

Mix.

The very pleasant taste and odor of this elixir, its freedom from color and chemical impurities, commends it for general use as a medicating vehicle.

RED ELIXIR.

| | | |
|---|---|---|
| Take of | Comp. Tinct. of Cochineal, . | $\frac{1}{2}$ fluid ounce. |
| | Simple Elixir, . . . . | 16 fluid ounces. |

Mix.

This is sometimes preferred as a simple elixir because of its beautiful color.

ELIXIR OF CALISAYA BARK.

| | | |
|---|---|---|
| Take of | Tinct. Cinchona, U. S. P., 1870, | 22 fluidrachms. |
| | Simple Elixir, | sufficient to make 16 fluid ounces. |

Mix and filter. This contains *the virtues* of two grains of Calisaya bark in one fluidrachm.

ELIXIR OF CALISAYA BARK AND IRON.

| | | |
|---|---|---|
| Take of | Elixir of Calisaya Bark, . . | 15 fluid ounces. |
| | Warm Distilled Water, . | 1 fluid ounce. |
| | Citrate of Iron, *soluble*, . . | 128 grains. |

Dissolve the iron in the warm water and add the elixir. Filter if necessary. Each fluidrachm of the unfiltered elixir contains one grain of the iron salt, and the virtues of nearly two grains of Calisaya bark.

COMPOUND ELIXIR OF CINCHONA.

| | | |
|---|---|---|
| Take of | Comp. Tinct. Cinchona, U.S.P., 1870, | 22 fluidrachms. |
| | Simple Elixir, . | sufficient to make 16 fluid ounces. |

Mix and filter. If not required for immediate use, this and also the Calisaya elixir should stand for about twelve hours before filtering.

### COMPOUND ELIXIR OF CINCHONA WITH IRON.

| | | |
|---|---|---|
| Take of | Compound Elixir of Cinchona, | 15 fluid ounces. |
| | Warm Distilled Water, . . | 1 fluid ounce. |
| | Citrate of Iron, *soluble*, . . | 120 grains. |

Mix. Proceed as for Elixir of Calisaya with Iron.

### ELIXIR OF CITRATE OF IRON.

| | | |
|---|---|---|
| Take of | Citrate of Iron, *soluble*, . . | 256 grains. |
| | Warm Distilled Water, . . | 1 fluid ounce. |
| | Simple Elixir, . . . . | 15 fluid ounces. |

Dissolve the iron in the warm water, and mix with the simple elixir. Filter.

### ELIXIR OF PYROPHOSPHATE OF IRON.

| | | |
|---|---|---|
| Take of | Pyrophosphate of Iron, . . | 256 grains. |
| | Warm Distilled Water, . | 1 fluid ounce. |
| | Simple Elixir, . . . | 15 fluid ounces. |

Make according to directions for Elixir of Citrate of Iron. This is the same medicinal strength as Prof. Diehl's formula.

### ELIXIR OF CITRATE OF BISMUTH.

Take of Citrate of Bismuth and Ammonium, 256 grains
Warm Distilled Water, . . 4 fluid ounces.
Water of Ammonia (drop by drop), sufficient.
Simple Elixir, sufficient to make 16 fluid ounces of finished elixir.

This is the same Bismuth strength as Prof. Diehl's formula, viz, two grains of citrate of bismuth and ammonium in each fluidrachm.

### ELIXIR OF PEPSIN.

| | | |
|---|---|---|
| Take of | Saccharated Pepsin, Scheffer's formula, | 256 grains. |
| | Sherry Wine, . . . . . | 14 fl. oz. |
| | Simple Syrup, . . . . . . | 2 fl. oz. |
| | Fluid Extract of Ginger, . . . | 25 drops. |

Dissolve the Pepsin in the wine, mix the fluid extract of ginger with the syrup, and mix altogether. Filter if necessary. Contains two grains of pepsin to the fluidrachm.

### ELIXIR OF VALERIANATE OF AMMONIUM.

| | | |
|---|---|---|
| Take of | Valerianate of Ammonium in crystals, | 256 grains. |
| | Compound Tinct. of Cochineal, . | $\frac{1}{2}$ fl. oz. |
| | Simple Elixir, . . . . | $15\frac{1}{2}$ fl. ozs. |

Dissolve the valerianate of ammonium in two ounces of the simple elixir, and carefully add water of ammonia until the solution is exactly neutral to test-paper. Mix with the balance of simple elixir, and then add the compound tincture of cochineal.

This is the formula of Prof. C. Lewis Diehl, with the exception of the simple elixir. Notwithstanding this preparation contains a larger quantity than usual of the valerianate of ammonium (two grains of the salt in each fluidrachm), yet its unpleasant taste and odor is effectually masked by the fragrance of the simple elixir.

ELIXIR OF VALERIANATE OF AMMONIUM WITH QUINIA.

| | | |
|---|---|---|
| Take of | Sulphate of Quinia, . . | 128 grains. |
| | Elixir of Valerianate of Ammonium, | 16 fl. oz. |

Mix. Filter if necessary. Sulphate of quinia is soluble in elixir of valerianate of ammonium to twice the quantity here ordered.

ELIXIR OF PYROPHOSPHATE OF IRON, QUINIA AND STRYCHNIA.

(*C. Lewis Diehl's formula.*)

He says: "This requires particular manipulation, which precludes the use of simple elixirs. The following formula, the result of concerted experiments of my friend, Mr. E. Scheffer, and myself, has been used by me since autumn, 1869, and I can recommend it as uniformly successful, when the manipulations are carefully conducted:

| | | |
|---|---|---|
| Take of | Sulphate of Quinia, . . | 60 grains. |
| | Strychnia, . . . . | 1 grain. |
| | Citric Acid, . . . . | 5 grains. |
| | Stronger Alcohol, . . . | 3 fluid ounces. |
| | Spirit of Orange, . . . | 80 minims. |
| | Syrup, . . . . . | 6 fluid ounces. |
| | Pyrophosphate of Iron, . . | ½ troy ounce. |
| | Distilled Water, . . . | 7 fluid ounces. |
| | Water of Ammonia, . . | suf. quantity. |

"Triturate the sulphate of quinia, strychnia and citric acid together, until minutely divided, then add the alcohol and spirit of orange. Warm the syrup slightly (to about 150° F.), and add to the turbid mixture, when, upon stirring, the mixture becomes clear. To this add the pyrophosphate of iron, previously dissolved in the distilled water; and finally, carefully add water of ammonia, drop by drop, until the elixir is perfectly neutral to test-paper; filter. The finished preparation has a greenish yellow color, a pleasant flavor of orange, and is permanent.

### BITTER WINE OF IRON.

*(James T. Shinn's formula, slightly modified.)*

We have had several years' experience with the following formula, and it has given entire satisfaction to prescriber, dispenser and consumer.

| | |
|---|---|
| Take of Sulphate of Cinchonia, . . . | 45 grains. |
| Sulphate of Quinia, . . . . | 15 grains. |
| Citric Acid, . . . . . . | 60 grains. |
| Citrate of Iron, *soluble*, . . . | 240 grains. |
| Con. Tinct. Fresh Sweet Orange peel, | 3 fl. ozs. |
| Distilled Water, . . . . . | 3 fl. ozs. |
| Sherry Wine, . . . . . . | 8 fl. ozs. |
| Syrup, . . . . . . . | 2 fl. ozs. |

Dissolve the sulphates and citric acid in two ounces of the water, and the iron in the remaining ounce of water; mix the two solutions, and add the other ingredients, previously well mixed together.

The only change from the original formula is in the kind and quantity of orange flavor, for which we claim an improvement.

### ELIXIR OF GENTIAN WITH IRON.

| | |
|---|---|
| Take of Extract of Gentian, . . . . | 128 grains. |
| Citrate of Iron, *soluble*, . . . | 128 grains. |
| Distilled Water, . . . . . | 1 fl. oz. |
| Simple Elixir, . . . . . | 15 fl. ozs. |

Dissolve the extract and iron in the water, *warmed*, and add the simple elixir; filter.

### ELIXIR OF BROMIDE OF POTASSIUM.

| | |
|---|---|
| Take of Bromide of Potassium, . . . . | 640 grains. |
| Red Elixir, . . . . . | 16 fl. ozs. |

Mix.

This contains five grains of the salt in each fluidrachm, and is given as a type. The red elixir does not seem to answer for the elixir bromide of calcium; caramel is a more suitable coloring substance for the calcium elixir. We prefer the simple elixir in this case, and to use no coloring substance.

SYRUP OF LICORICE ROOT.

| | | |
|---|---|---|
| Take of | select Licorice Root, in moderately coarse powder, . | 4 troy ounces. |
| | Diluted Alcohol, . . . | sufficient quantity. |
| | Sugar, . . . . . | 12 troy ounces. |

Moisten and pack in a conical percolator; macerate for 12 hours, percolate to exhaustion. Place the tincture over a water bath until reduced to ten fluid ounces, filter, and then add the sugar; lastly, sufficient distilled water to make sixteen fluid ounces of finished syrup.

The syrup of licorice root, when carefully prepared, is more effectual and more convenient for masking the bitterness of quinia than is the very popular "compound elixir of taraxacum," and being free from the stimulating influence of alcohol, which is present in the elixir, is well adapted for children. The proper proportions will be one grain of quinia (any salt of it) to the fluidrachm, and if those for whom quinia is ordered will take the precaution to chew a small quantity of licorice root previous to taking the quinia mixed with the syrup of licorice, in the proportions here recommended, scarcely any bitterness will be observed. As a matter of course, acids mixed with quinia and licorice syrup will immediately develop the bitter taste.

It has of late become fashionable to use glycerin as an antiseptic and solvent in elixirs, as well as other compounds of pharmacy, but our aversion to the general use of glycerin for internal administration, for various reasons, has prevented its introduction in our formulas.

The results of our investigations of liquid pepsin preparations will not warrant the introduction of more than the one formula, which is really a wine of pepsin, and has been found useful in many cases.

Mr. A. P. Brown stated that he had made some of these elixirs, and found them to answer their intended purposes well; the simple and red elixirs can be used as vehicles, and may be diluted with water without becoming turbid.

